# Exploring pharmacokinetic variability of palbociclib in HR+/HER2- metastatic breast cancer: a focus on age, renal function, and drug–gene interactions

**DOI:** 10.3389/fphar.2024.1420174

**Published:** 2024-09-06

**Authors:** Elena Peruzzi, Bianca Posocco, Lorenzo Gerratana, Margherita Nuti, Marco Orleni, Sara Gagno, Elena De Mattia, Fabio Puglisi, Erika Cecchin, Giuseppe Toffoli, Rossana Roncato

**Affiliations:** ^1^ Experimental and Clinical Pharmacology, Centro di Riferimento Oncologico di Aviano (CRO), IRCCS, Aviano, Italy; ^2^ Department of Medical Oncology, Centro di Riferimento Oncologico di Aviano (CRO), IRCCS, Aviano, Italy; ^3^ Department of Medicine (DMED), University of Udine, Udine, Italy; ^4^ Doctoral School in Pharmacological Sciences, University of Padua, Padova, Italy

**Keywords:** palbociclib, metastatic breast cancer, minimum plasma concentration, pharmacokinetic variability, pharmacokinetic covariate, drug-drug interactions, drug-gene interaction, drug-drug-gene interactions

## Abstract

Palbociclib, an oral inhibitor of cyclin-dependent kinase 4 and 6, is approved for the treatment of metastatic breast cancer. This study investigated the influence of diverse clinical and biological factors—age, renal function, genetic variations, and concomitant medications (*pharmacokinetic covariates*)—on palbociclib pharmacokinetics. Employing a validated LC-MS/MS method, we analyzed the minimum plasma concentrations (C_trough_) of palbociclib in 68 women and determined the percentage deviations from the median C_trough_ for each dosage group. Variations in a panel of absorption, distribution, metabolism, and excretion (ADME) genes were assessed using end-point allele-specific fluorescence detection and pyrosequencing. Two distinct patient cohorts were defined based on median values of age, creatinine, and eGFR, which exhibited statistically significant differences in percentage deviations (*p* = 0.0095, *p* = 0.0288, and *p* = 0.0005, respectively). Homozygous carriers of the *PPARA* variants displayed larger positive percentage deviations than the other group (*p* = 0.0292). Similarly, patients concurrently taking CYP3A and P-glycoprotein inhibitors alongside anticancer therapy exhibited significant variations (*p* = 0.0285 and *p* = 0.0334, respectively). Furthermore, exploring the drug–drug–gene interactions between inhibitors of CYP3A and P-glycoprotein with their respective genetic variants revealed two patient groups with statistically different percentage deviations (*p* = 0.0075, *p* = 0.0012, and *p* = 0.0191, respectively). These results could help address cases where pharmacokinetic covariates or subclinical conditions impair palbociclib adherence or response, aiming to offer tailored dosing strategies or monitoring for individual patients.

## 1 Introduction

Hormone receptor-positive (HR+) and human epidermal growth factor receptor 2-negative (HER2–) metastatic breast cancers account for a substantial proportion of all breast cancer cases. The evolution of targeted therapies has significantly altered the treatment paradigm for this subgroup, with a notable shift toward preferring endocrine therapy, integrated with cyclin-dependent kinase 4 and 6 (CDK4/6) inhibitors, over traditional chemotherapy (Bonotto et al., 2017; Giuliano et al., 2019).

Palbociclib, a selective CDK4/6 inhibitor, has emerged as a pivotal treatment in managing HR+/HER2– metastatic breast cancer. Approved by the Food and Drug Administration (FDA) and the European Medicines Agency (EMA) in 2015, palbociclib is administered at the starting dose of 125 mg once daily for 21 days, followed by 7 days off for a 28-day cycle (EMA, 2024; FDA, 2024b). Its pharmacokinetics reveal a low bioavailability (approximately 46%), a maximum concentration reached between 6- and 12-h post-ingestion, and a steady state achieved within 8 days of daily dosing. Its metabolism primarily occurs through cytochrome P450 (CYP) 3A isoform and sulfotransferase (SULT) 2A1. The major route of excretion is through feces, accounting for 74.1% of the dose, while approximately 17.5% of the dose is recovered in urine (Center for Drug Evaluation and Research, 2014). Additionally, palbociclib has been described, in *in vivo* and *in vitro* studies, as a substrate of the breast cancer resistance protein (BCRP) and the efflux transporter P-glycoprotein (P-gp) (Braal et al., 2021).

The pharmacokinetic profile of palbociclib, like other oral targeted therapies, is characterized by intra- and inter-individual variability, as indicated by a coefficient of variation of 41%–59% in trough concentrations (Groenland et al., 2020). This variability, influenced by covariates such as patient-specific characteristics (age, gender, race, weight, hormonal status, renal and hepatic function, and pharmacogenetics), diet, comorbidities, treatment adherence, and concomitant medications, underscores the need for a deeper understanding.

Previous research has shed light on several aspects of palbociclib pharmacokinetics; however, gaps remain in comprehensively understanding the influence of various covariates (Klümpen et al., 2011; Leenhardt et al., 2022; Roncato et al., 2022; Roncato et al., 2023; Peruzzi et al., 2023a).

This study aims to bridge these knowledge gaps by examining the clinical and biological factors that significantly impact the pharmacokinetics of palbociclib in patients with HR+/HER2- metastatic breast cancer. Additionally, given the higher likelihood of drug–drug interactions (DDIs) in cancer patients on multiple treatments, understanding the interactions resulting from concomitant medication is crucial. This includes pharmacokinetic interactions from the co-administration of inhibitors or inducers of enzymes and transporters involved in palbociclib’s pharmacokinetic profile, a critical aspect of all oral targeted therapies. Furthermore, this study also seeks to analyze the complex interplay between drug–gene interactions (DGIs) (Peruzzi et al., 2023b) and DDIs by assessing drug–drug–gene interactions (DDGIs), which allows us to evaluate the phenotype of absorption, distribution, metabolism, and excretion (ADME) enzymes and transporters based on both genetic variations and concomitant medications use.

Therefore, in a cohort of women with metastatic breast cancer treated with palbociclib, we determined the minimum palbociclib plasma concentration at the steady state (C_trough_) and investigated the influence of the aforementioned factors to highlight the clinical relevance of these factors in optimizing the therapeutic effect and improving patient safety by minimizing variability.

## 2 Materials and methods

### 2.1 Study design and patients

This study includes patients treated with palbociclib (days 1–21 of each 28-day cycle) affected by HR+/HER2- metastatic breast cancer and retrospectively selected from the cohort of the CRO–Aviano integrated pharmacological counseling program (Roncato et al., 2022) approved by the local ethics committee (Comitato Etico Unico Regionale del Friuli Venezia Giulia—CEUR) and conducted in accordance with the principles of the Declaration of Helsinki in its latest version (protocol ID: CRO 2022–14, approval date 12 April 2022; Parere-CEUR-2022-Os-65). Written informed consent was obtained from the patients.

The eligibility criteria were as follows: (i) patients with a diagnosis of HR+/HER2- metastatic breast cancer; (ii) patients who underwent treatment with palbociclib as either first- or second-line therapy, combined with aromatase inhibitors or fulvestrant; (iii) patients with available clinical data; (iv) patients with at least one blood sample collected at any palbociclib dose level (125 mg, 100 mg, or 75 mg) for evaluating the C_trough_.

### 2.2 Palbociclib plasma concentration

Palbociclib C_trough_ was retrieved by plasma sample centrifuged at 16,200 *g* for 10 min at 4°C in whole blood EDTA tubes and analyzed with a validated LC-MS/MS method (Posocco et al., 2020; Posocco et al., 2024). To assess C_trough_, samples should be taken at a steady state and 24 h after the last dose. Samples collected within a 22–26 h window after the last intake of palbociclib were included. The median C_trough_ value and the coefficient of variation (CV, in percentage) were calculated for each palbociclib dose (125 mg, 100 mg, and 75 mg). For each patient with more than one sample at the same dosage, the mean of the measured C_trough_ values was considered.

To comprehensively analyze the C_trough_ values associated with various doses of palbociclib, we calculated the percentage deviation of each C_trough_ from the specific median C_trough_ of each dosage level. This newly derived parameter, hereafter referred to as the “percentage deviations,” allowed for a detailed and comparative understanding of the pharmacokinetic behavior of palbociclib across its different therapeutic dosages. Positive percentage deviations represent C_trough_ values higher than the dose-specific median, while negative values denote a C_trough_ below the corresponding median. The magnitude of the absolute percentage deviations reflects the degree of deviation from the specific median C_trough_.

### 2.3 Pharmacokinetic covariates

Several factors were considered to analyze the palbociclib pharmacokinetic variability found in this selected case study. Such factors were classified as “pharmacokinetic covariates” and include patient clinical and pharmacological characteristics: age, hormonal status, treatment setting, hepatic and renal function, hematologic values, polymorphisms of ADME gene, and concomitant medications. All these factors were retrieved from the electronic medical record and/or directly from the patients at the time of sampling. Except for the pharmacogenetics, clinical characteristics were collected at baseline and at the time of sampling or, if not available, at the earliest time of sampling (within 2 weeks).

#### 2.3.1 Clinical and biological characteristics

Only clinical–biological data associated to the sampling were considered for the evaluation of pharmacokinetic interactions. In instances where mean C_trough_ values were considered, the average of the corresponding clinical and biological characteristics was also computed, provided that multiple data points were available. For hematologic data, white blood cell (WBC), absolute neutrophil count (ANC), and hemoglobin (Hgb) were evaluated. Aspartate and alanine aminotransferase (AST and ALT) and total bilirubin (BILT) were used to assess liver function, while creatinine and the calculated estimated glomerular filtration rate (eGFR) were used to assess renal function. The eGFR was calculated using the following formula derived from the Modification of Diet in Renal Disease (MDRD) study (Beck, 2023):

$$eGFR=186\times \left({creatinine}^{-1.154}\right)\times \left({age}^{-0.203}\right)\times 0.742.$$

#### 2.3.2 ADME gene polymorphisms

Polymorphism analyses of the ADME gene were performed by the SNPline PCR Genotyping System platform using Kompetitive allele–specific assays (LGC Genomics, Hoddesdon, UK) following DNA extraction from blood samples. The DNA extraction was done using the BioRobot EZ1 system workstation (Qiagen N.V., Germany) and the EZ1 DNA Blood 200 µL Kit. The analyzed polymorphisms were *CYP3A* (e.g., CYP3A4*22, rs35599367; CYP3A5*3, rs776746; CYP3A5*6, rs10264272; CYP3A5*7, rs41303343), nuclear receptor influencing CYP450 (e.g., POR*28, rs1057868; *PPARA* rs4253728; *PPARA* rs4823613; *PXR* rs2472677; *PXR* rs3814055; *VDR* rs11568820; *VDR* rs1544410; *VDR* rs4516035), and transporters (e.g., *ABCB1* c.1236C>T, rs1128503; *ABCB1* c.3435C>T, rs1045642; *ABCG2* c.421C>A, rs2231142). In addition, the triallelic polymorphisms of *ABCB1* 2677G>T/A (rs2032582) were analyzed using pyrosequencing technology from PyroMark Q48 (Qiagen, Hilden, Germany) to achieve triallelic discrimination. Positive and negative control samples were included in each analysis.

##### 2.3.2.1 Metabolizer status attribution

Each patient was given a *CYP3A* metabolizer status. Poor metabolizers (PM) were carriers of the CYP3A4*1/*22 or *22/*22 and CYP3A5*3/*3 diplotypes. Intermediate metabolizers (IM) were carriers of the CYP3A4*1/*1 and CYP3A5*3/*3 diplotypes as well as the CYP3A4*1/*22 or *22/*22 and CYP3A5*1/*3 or *1/*1 diplotypes. Lastly, normal metabolizers (NM) were carriers CYP3A4*1/*1 and CYP3A5*1/*3 or *1/*1 diplotypes (Lloberas et al., 2017; Sanchez Spitman et al., 2017).

#### 2.3.3 Concomitant medication

All recorded concomitant medications were classified according to the therapeutic class and the anatomical–therapeutic–chemical (ATC) classification (last update 23 January 2023). To analyze the influence of concomitant medications on the pharmacokinetic variability of palbociclib, we first examined the influence of the number of concomitant medications each patient was taking concomitantly with oncology therapy and at the time of sampling. Patients with zero or one co-medication were compared with patients with 2–4 concomitant medications and with patients who had five or more concomitant medications. Next, the influence of co-administering at least one drug that acts as an inhibitor or inducer of CYP3A and P-gp, and BCRP was analyzed. Such inhibitors or inducers were classified based on four different sources: US Food and Drug Administration Pharmacological Review and Drug Interactions Flockhart Table and online sources “UptoDate” and “Cancer Liverpool Interactions” (Clinical Pharmacology School of Medicine, 2024; FDA, 2024a; UpToDate Free, 2023). Patients taking any type of inhibitor or inducer (weak, moderate, or strong) were compared with those not taking any them. Finally, the impact of the most common concomitant drugs recorded in the selected case study was also investigated.

#### 2.3.4 Drug–drug–gene interactions

To evaluate the impact of DDGI on palbociclib C_trough,_ patients with genetic variations that reduce the functionality of palbociclib ADME enzymes and transporters and/or those taking inhibitors of these enzymes and transporters were grouped together and compared to others.

### 2.4 Statistical analysis

Quantitative variables were reported as the number of observations (n), frequency (%), median, and interquartile range (IQR), while qualitative variables were described as the number of observations (n) and frequency (%). Then, the impact of each pharmacokinetic covariate was evaluated by a non-parametric Mann–Whitney or Kruskal–Wallis test. The plotted graphs report the median and the IQR for each compared group. Regarding ADME gene polymorphisms, the best-fitting genetic model (dominant or recessive) was used to perform the association analyses. All statistical analyses and graphs were performed using GraphPad Prism 9.2.0 version, and the significance level was set at 0.05.

## 3 Results

### 3.1 Patients and palbociclib plasma concentration

**FIGURE 1:**
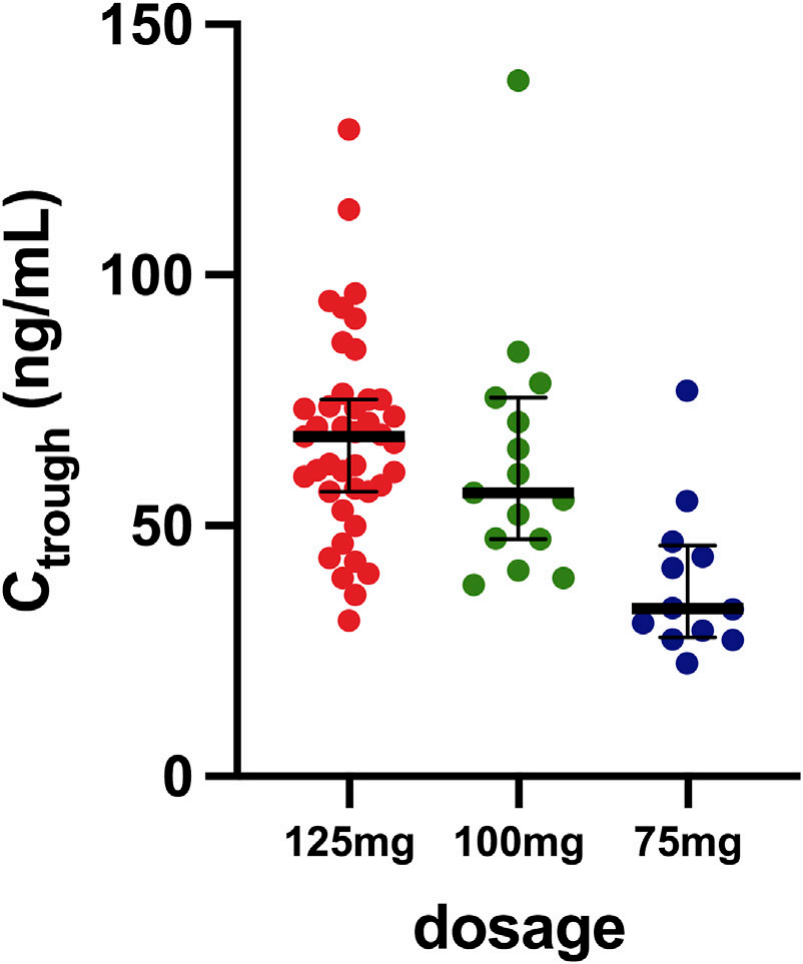
Distribution of samples based on the different dosages. The horizontal bar represents the median with the IQR, while the dots represent values of minimum plasma concentrations measured.

In this study, a total of 68 female patients diagnosed with HR+/HER2- metastatic breast cancer were enrolled, receiving treatment with palbociclib at the National Cancer Institute, IRCCS, Aviano. The baseline clinical characteristics of these patients are delineated in Table 1. Predominantly, the cohort comprised individuals undergoing first-line treatment with letrozole as the primary anti-hormonal therapy. Among the 68 patients, 45 (66%) contributed a single sample for the assessment of palbociclib C_trough_, while 23 (34%) provided multiple samples (two or three). Figure 1 shows the collected samples categorized based on the dosage. The median C_trough_ values observed were 68 ng/mL (IQR 57–75; CV 30%) for the 125 mg dosage, 57 ng/mL (IQR: 47–76; CV: 40%) for the 100 mg dosage, and 33 ng/mL (IQR: 28–46; CV: 40%) for the 75 mg dosage.

**TABLE 1:** Description of baseline characteristics.

Patient characteristics	Tot n (%)
Total patients enrolled	68
Age at enrollment (years)	
Median (IQR)	65 (54.0–71.3)
Hormonal status	
Pre-menopausal status	16 (23.5)
Post-menopausal status	52 (76.5)
Anti-hormonal therapy	
Letrozole	41 (60.3
Fulvestrant	26 (38.2)
Anastrozole	1 (1.5)
Treatment line	
First line	51 (75.0)
Second line	17 (24.5)
Total samples collected	91
Samples at 125 mg	54 (59.3)
Samples at 100 mg	21 (23.1)
Samples at 75 mg	16 (17.6)

Abbreviations: IQR, interquartile range.

### 3.2 Clinical and biological data and palbociclib plasma concentration

**FIGURE 2:**
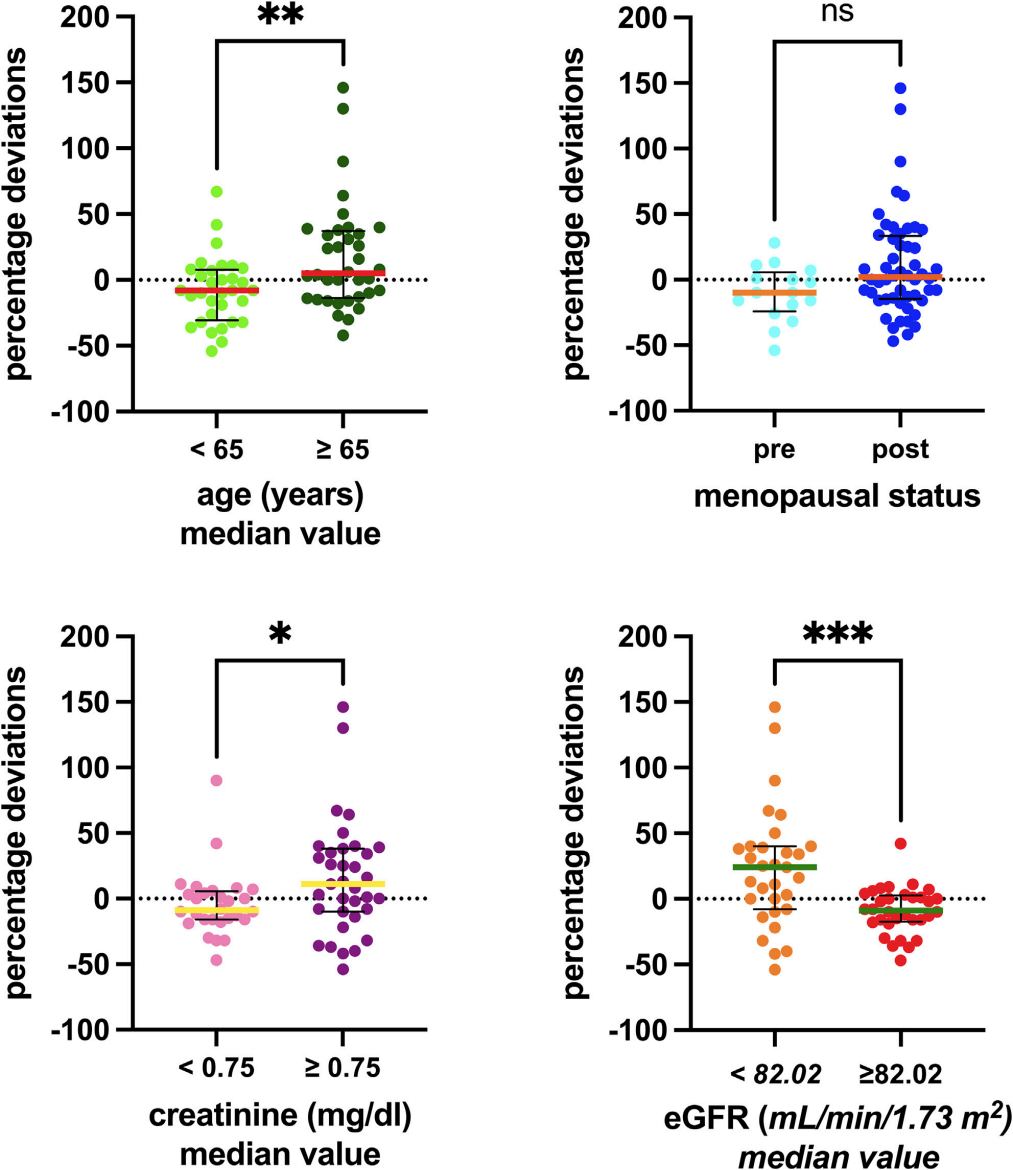
Associations between the clinical–biological factors recorded and the percentage deviations. The box plot depicts the median (horizontal bar) and the IQR. The dots represent the percentage deviations. Abbreviations: ns, not significant; eGFR, estimated glomerular filtration rate.

Table 2 comprises the available clinical and biological parameters recorded for each patient’s sample. Figure 2 shows the results of the Kruskal–Wallis tests, examining the association between the percentage deviation and selected clinical–biological factors. Patients aged 65 years and older demonstrated a significantly more pronounced positive percentage deviation than those under 65 years, with median values for the older group being 13 percentage points higher (*p* = 0.0095). Although a similar trend was observed in relation to the menopausal status, it did not reach statistical significance (*p* = 0.0581). Patients with creatinine levels above 0.75 mg/dL had 20 percentage-point higher deviations than those with lower levels (*p* = 0.0288). Similarly, patients with eGFR below 82.02 mL/min/1.73 m^2^ had 33 percentage-point higher deviations than those with higher eGFR (*p* = 0.0005). Conversely, no significant differences were observed for other factors such as ANC, WBC, hemoglobin, ALT, AST, and bilirubin.

**TABLE 2:** Description of biological factors collected.

Chemical values	Tot n^a^	Median value (IQR)
Renal values		
Creatinine (mg/dL)	63	0.75 (0.7–0.9)
eGFR (mL/min/1.73 m ^ 2)	63	82.02 (71.0–95.2)
Hepatic values		
AST (U/L)	63	19.0 (14.5–24.0)
ALT (U/L)	63	14.0 (10.0–20.0)
BILT (mg/dL)	62	0.40 (0.3–0.5)
Hematologic values		
WBC (x10 ^ 3 μL)	66	3.16 (2.5–3.7)
Hgb (g/dL)	65	12.10 (11.5–12.8)
ANC (x10 ^ 3 μL)	66	1.37 (1.1–1.6)

^a^ Available chemical values are reported at the time of blood sample collection or at the immediately preceding blood test.

Abbreviations: IQR, interquartile range; eGFR, estimated glomerular filtration rate; AST, aspartate aminotransferase; ALT, alanine aminotransferase; BILT, total bilirubin; WBC, white blood cells; Hgb, hemoglobin; ANC, absolute neutrophil count.

### 3.3 Pharmacogenetic data and palbociclib plasma concentration

**FIGURE 3:**
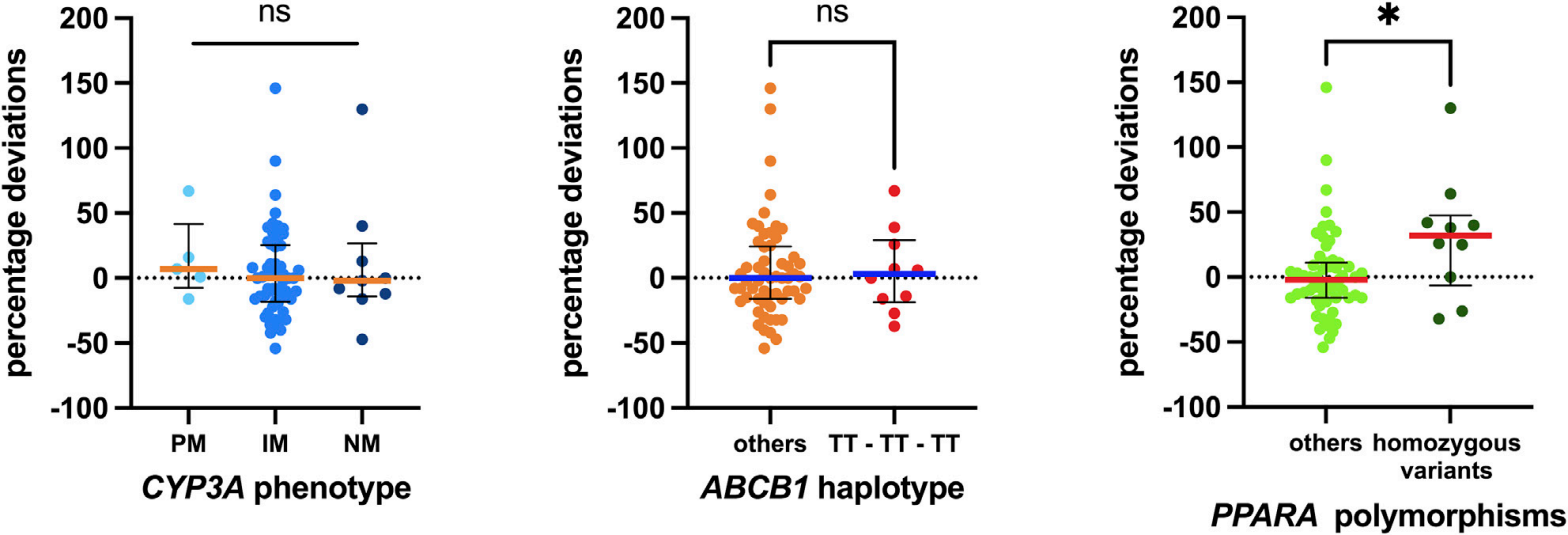
Associations between pharmacogenetic data detected and the percentage deviations calculated. Regarding *CYP3A*, the status of the three metabolizers was evaluated. For *ABCB1*, the 1236T-3435T-2677T/A haplotype was considered, and the homozygous carriers were compared with all others. Finally, homozygous carriers of both variant polymorphisms of *PPARA* were compared with others. The box plot depicts the median (horizontal bar) and the IQR. The dots represent the percentage deviations. Abbreviations: ns, not significant;, PM, poor metabolizer; IM, intermediate metabolizer; NM, normal metabolizer.

Sixteen single-nucleotide polymorphisms (SNPs) were tested, and their distributions across genotypes are reported in Supplementary Table S1, while Figure 3 illustrates the associations investigated. Although complete SNP data were not obtainable for all patients, those analyzed did not show significant deviation from the Hardy–Weinberg equilibrium. Notably, significant associations were observed only for two polymorphisms in the ADME gene, *PPARA* (rs4253728 and rs4823613), which are in linkage disequilibrium. Patients with homozygous variant genotypes for both polymorphisms exhibited significantly greater positive percentage deviations, with a median deviation 34 percentage points higher than those with wild-type or heterozygous genotypes (*p* = 0.0292).

### 3.4 Concomitant medication and palbociclib plasma concentration

**FIGURE 4:**
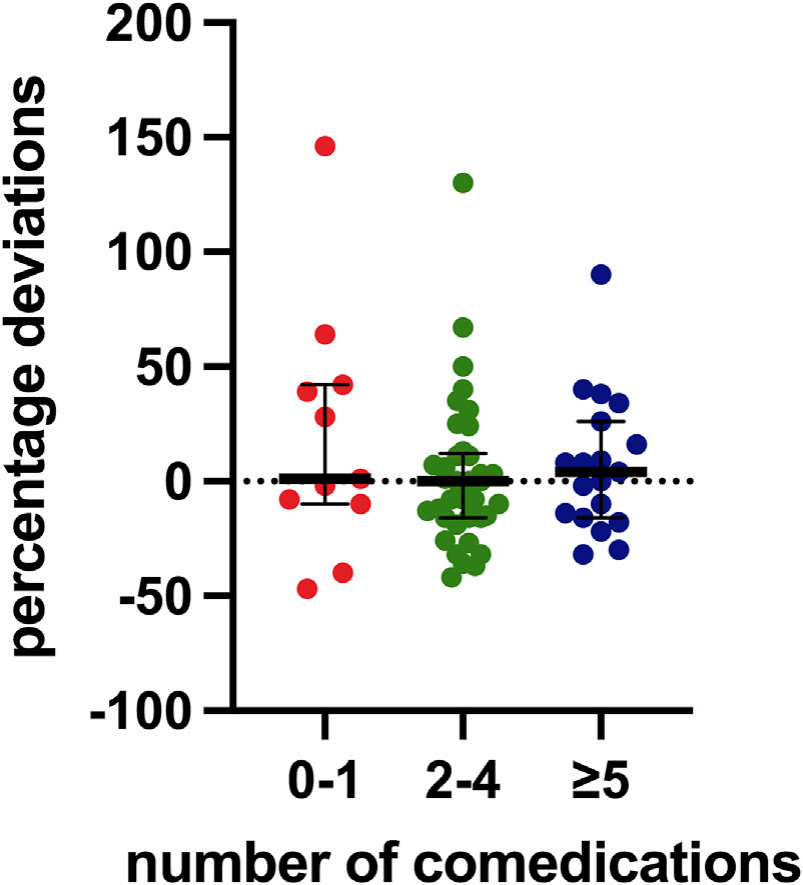
Distribution of percentage deviation values based on the number of concomitant medications. The box plot depicts the median (horizontal bar) and the IQR. The dots represent the percentage deviations.

Regarding concomitant medications, all patients were on at least one additional drug, with an average of four per patient. Ten patients (14.7%) were on a single concomitant medication, while 37 (54.4%) were taking between two and four medications, and 19 (27.9%) were on five or more. Figure 4 presents these data.

**FIGURE 5:**
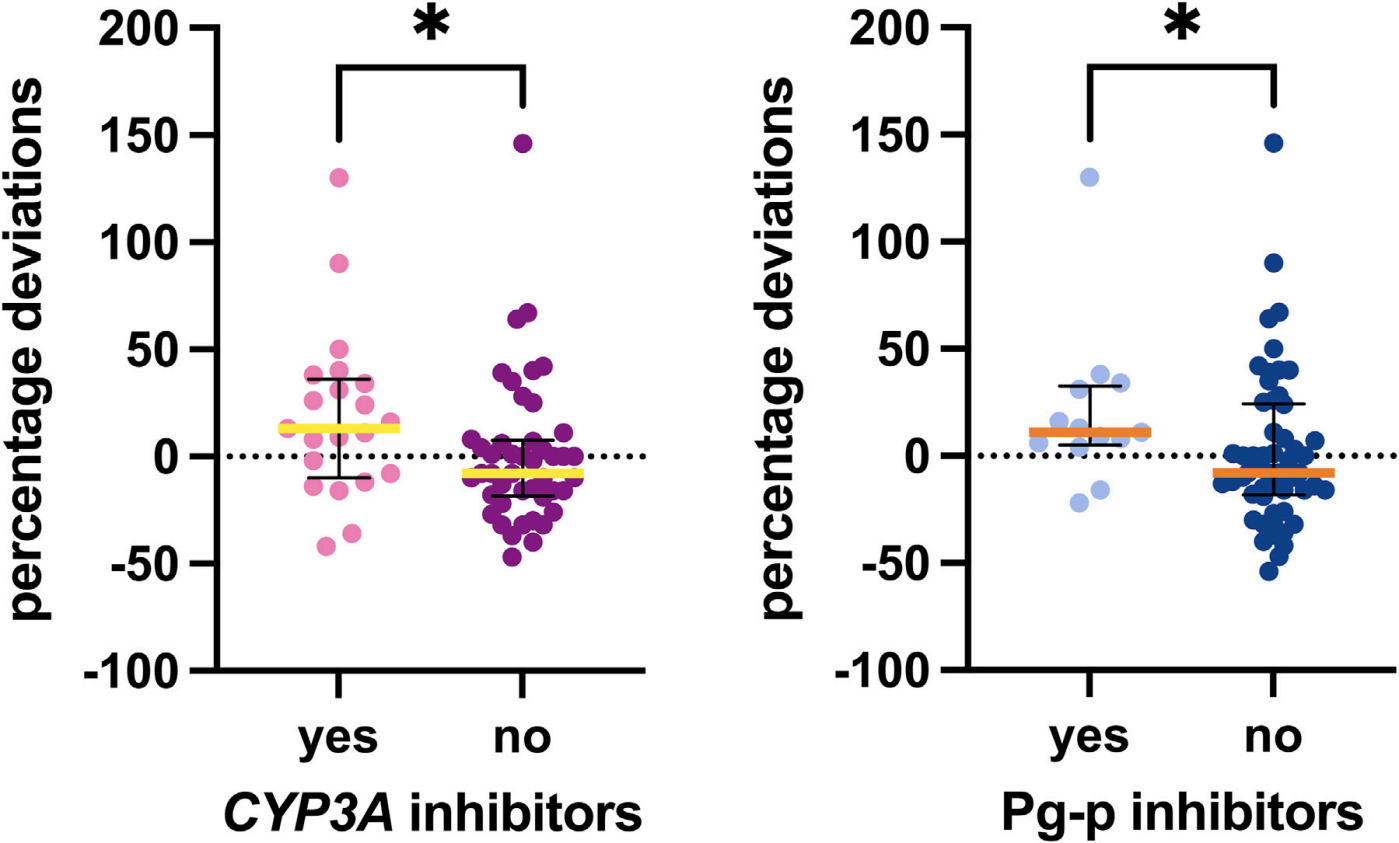
Associations between inhibitors detected and the percentage deviations. The box plot depicts the median (horizontal bar) and the IQR. The dots represent the percentage deviations.

Our analysis specifically focused on inhibitors and inducers of CYP3A, P-gp, and BCRP. Only a limited number of weak and moderate inhibitors of CYP3A and P-gp were recorded, with just one patient taking a P-gp inducer. No BCRP inducers or inhibitors were found. Associations identified with these elements are shown in Figure 5. Patients taking weak or moderate inhibitors were grouped in the “yes” column. Notably, a statistically significant difference was observed with CYP3A and P-gp inhibitors, with median values for the group taking at least one CYP3A inhibitor being 21 percentage points higher and those for the group taking at least one P-gp inhibitor being 19 percentage points higher than those not taking these inhibitors (*p* = 0.0285 and *p* = 0.0334, respectively).

**FIGURE 6:**
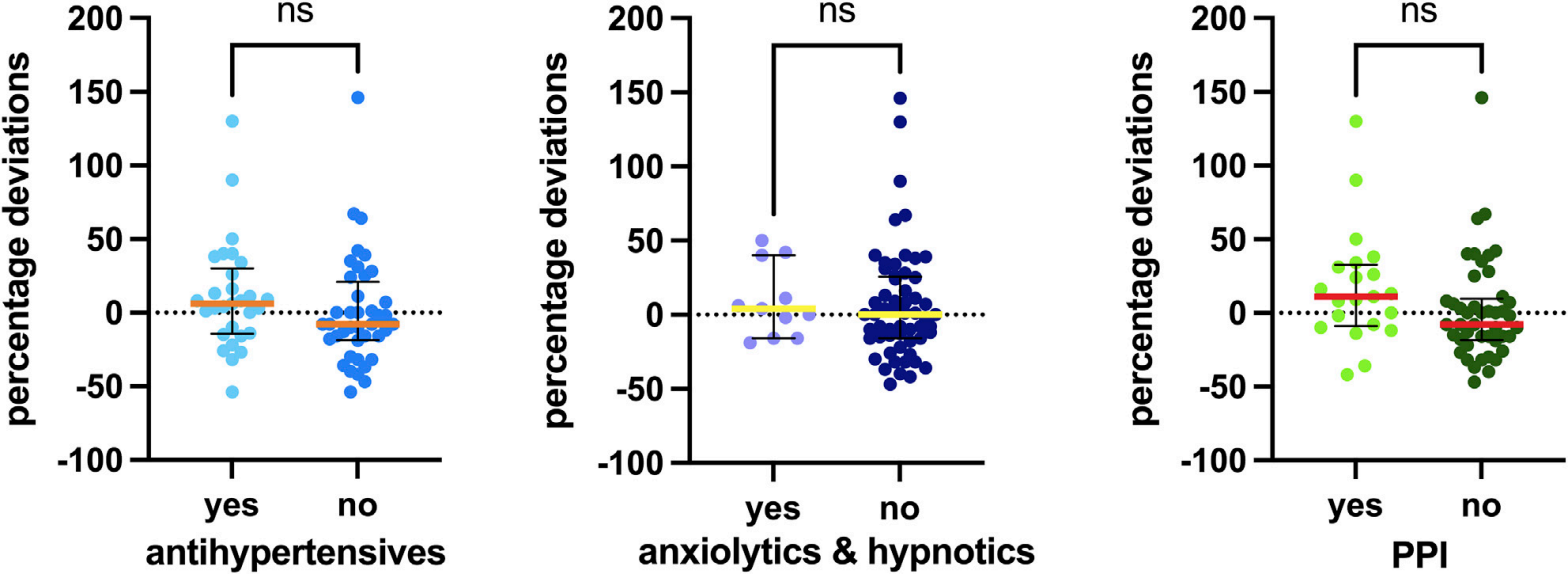
Associations between concomitant medications detected and the percentage deviation. The box plot depicts the median (horizontal bar) and the IQR. The dots represent the percentage deviations. Abbreviations: ns, not significant; PPI, proton pump inhibitor.

Additionally, Figure 6 illustrates the associations with the most frequently recorded concomitant medications in our study: antihypertensives, anxiolytics/hypnotics (especially benzodiazepines), and proton pump inhibitors (PPIs). No statistically significant difference was observed in palbociclib exposure among patients taking these types of concomitant medications.

### 3.5 Drug–drug–gene interactions and palbociclib plasma concentration

**FIGURE 7:**
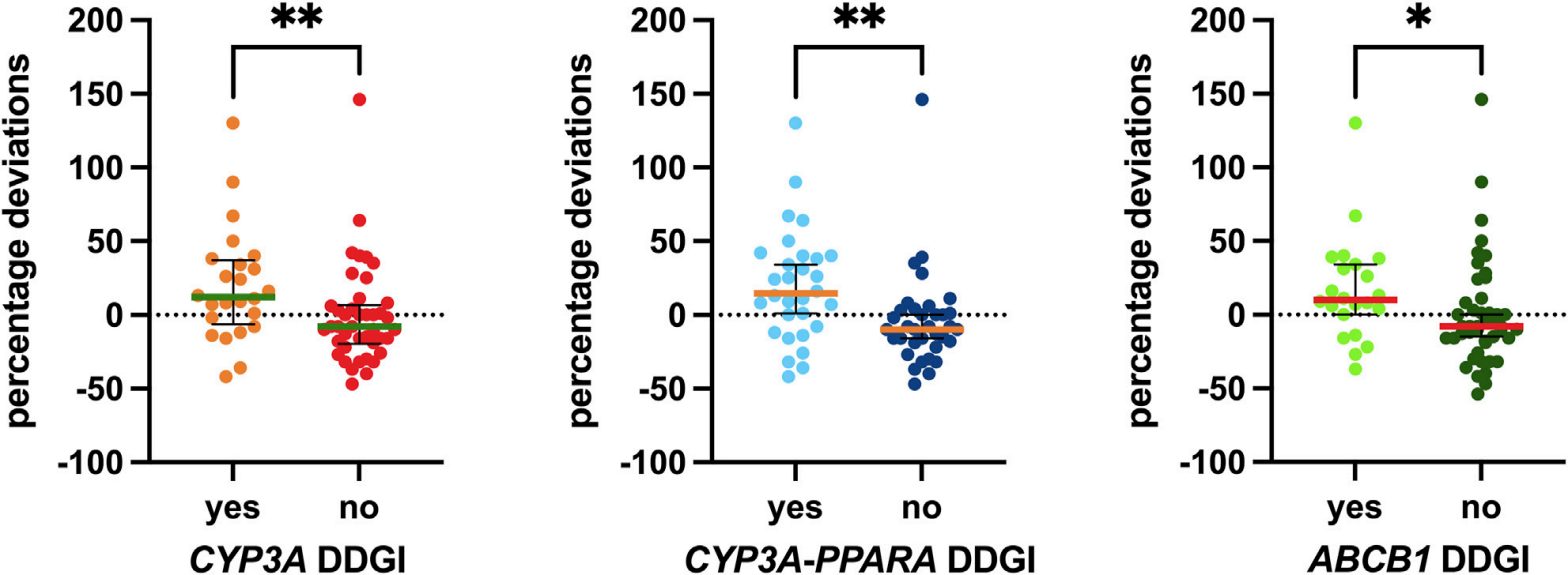
Associations between DDGI and the percentage deviations. The box plot depicts the median (horizontal bar) and the IQR. The dots represent the percentage deviations. Abbreviation: DDGI, drug–drug–gene interactions.

The DDGI analyses are shown in Figure 7. The first analysis, “*CYP3A* DDGI,” compares patients exhibiting a PM *CYP3A* phenotype and/or those taking CYP3A inhibitors as concomitant medications (grouped in the “yes” column) with other patients. In the second analysis, the “yes” column includes patients with a PM *CYP3A* phenotype, carriers of homozygous variants in both *PPARA* polymorphisms, and/or those using CYP3A inhibitors. Finally, a similar analysis was conducted for *ABCB1*, comparing patients carrying mutated *ABCB1* haplotypes and/or those taking P-gp inhibitors as concomitant medications (grouped in the “yes” column) with other patients.

Specifically, the median values for the group with *CYP3A* DDGI were 20 percentage points higher than those without. For the group with *CYP3A-PPARA* DDGI, the median values were 24.5 percentage points higher, and for the group with *ABCB1* DDGI, the median values were 18 percentage points higher than those without these DDGIs. All three associations were statistically significant (*p* = 0.0075, *p* = 0.0012, and *p* = 0.0191, respectively).

## 4 Discussion

The findings of this study offer significant insights into the pharmacokinetic variability of palbociclib in patients with HR+/HER2- metastatic breast cancer, particularly in light of its complex metabolism and excretion pathways. The study’s detailed examination of palbociclib pharmacokinetic variability highlights the significance of patient-specific factors like age, renal function, genetic polymorphisms, DDIs, and DDGIs in influencing drug exposure, offering findings that might be crucial for a deeper understanding of pharmacokinetic variability.

The median C_trough,_ calculated within our study group treated with the standard 125 mg dose of palbociclib aligns with the mean value of this parameter, as reported in the FDA’s Clinical Pharmacology Review. The reported palbociclib C_trough_ value stands at 61 ng/mL with a CV of 42%. This characterization was conducted in both healthy subjects and patients with solid tumors, including those with advanced breast cancer (Center for Drug Evaluation and Research, 2014). Additionally, we provided median C_trough_ values for two-step dose reductions, specifically 100 mg and 75 mg, showcasing decreases of 16% and 51%, respectively, from the median C_trough_ calculated at the standard dosage.

Regarding the covariates analyzed, our analysis revealed a notable age-related difference in palbociclib exposure. Patients older than 65 years exhibited a higher median palbociclib exposure (expressed as a percentage deviation from the median C_trough_). This finding aligns with the understanding that physiological changes associated with aging can affect drug metabolism and distribution, suggesting a potential need for age-adjusted dosing strategies in older patients.

The study also identified significant associations between palbociclib exposure and renal function markers such as creatinine levels and eGFR. Given that eGFR is calculated using age and creatinine levels, the significant association of eGFR with percentage deviation may be influenced by these underlying factors. This is in line with findings by Leenhardt F. et al., who reported that palbociclib C_trough_ was higher than the median values in older patients and those with reduced kidney function (Leenhardt et al., 2022). These findings suggest that even subclinical alterations in renal function, potentially associated with age, may still be important factors to consider in the dose adjustment and monitoring of palbociclib-treated patients. This is a noteworthy consideration, especially given the drug’s excretion profile, which indicates that palbociclib is predominantly excreted in the feces.

Our genetic analysis revealed a noteworthy association between specific *PPARA* gene polymorphisms and palbociclib exposure, underscoring the potential role of genetic factors in individual variability in drug response. Carriers of homozygous variant alleles for both *PPARA* gene polymorphisms exhibited a higher median palbociclib exposure (expressed as a percentage deviation from the median C_trough_) than patients who were either heterozygous or carriers of the wild-type alleles for both variants. Therefore, this insight brings to light the key role of nuclear receptors, which are known to be involved in the regulation and transcription of the cytochrome gene and to explain a percentage of the CYP3A4 activity variation.

Our study further explored the impact of concomitant medications on palbociclib exposure. While the drugs assessed in our study were not potent cytochrome inhibitors, the significant increase in exposure observed in patients receiving CYP3A and P-gp inhibitors underscores the necessity of incorporating DDIs into patient evaluations. This is essential to accurately ascertain the metabolizer status for ADME enzymes in conjunction with DGIs. This consideration becomes increasingly relevant due to the prevalent use of CYP3A substrates among cancer patients, both as part of concomitant medications and within oral anticancer agents. Leenhardt F. et al. also provided evidence of a higher palbociclib C_trough_ in patients taking at least one CYP3A4 or P-gp inhibitor (Leenhardt et al., 2022). Furthermore, our data reveal that when considering various factors in combination, particularly the use of CYP3A inhibitors and a reduced-function phenotype (PM) of the *CYP3A* gene, there is a notable increase in palbociclib exposure (expressed as a percentage deviation from the median C_trough_) compared to other groups. This was also distinctly observed in individuals with homozygous variants of the *PPARA* polymorphisms.

**FIGURE 8:**
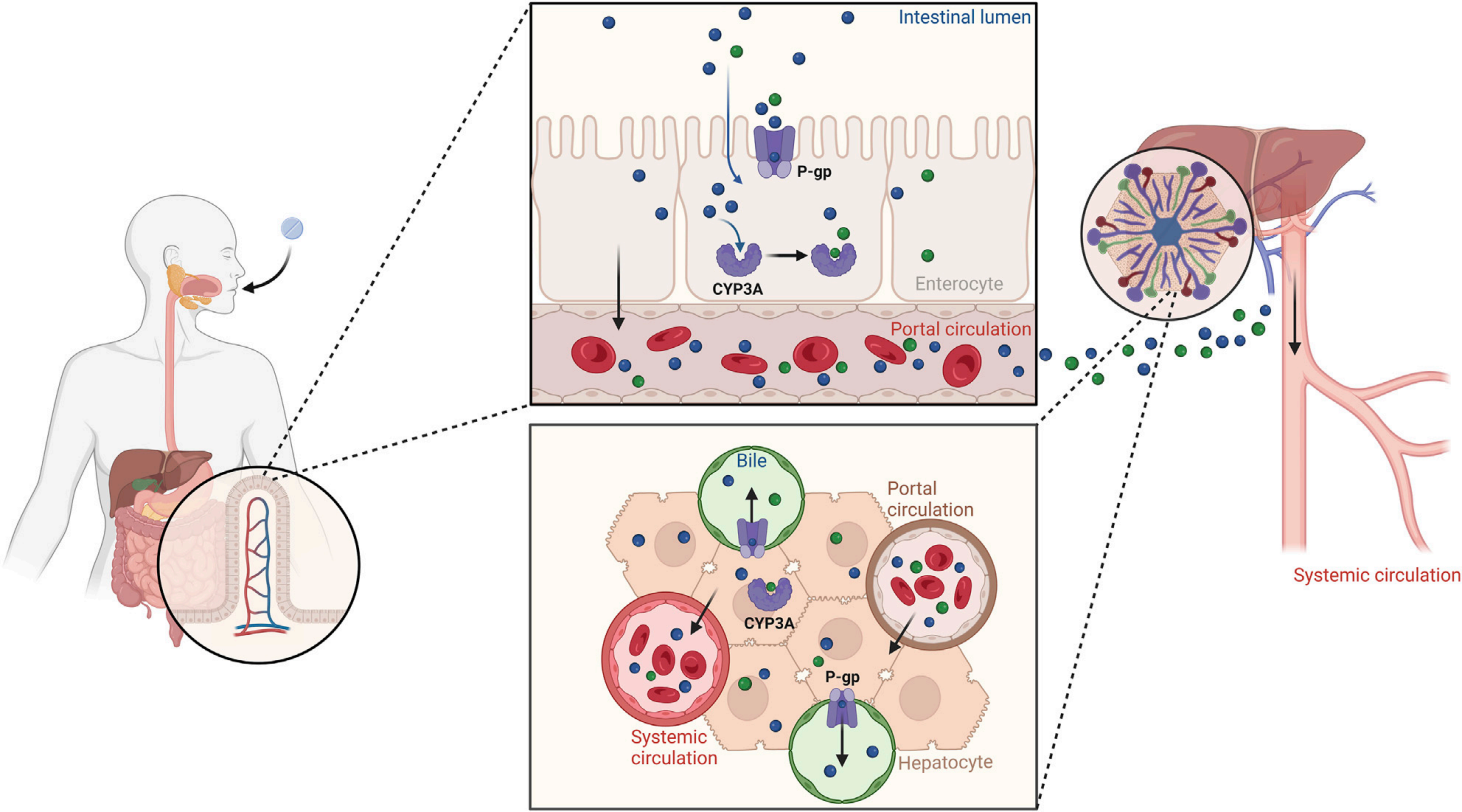
Illustration of the roles of P-gp and CYP3A in the palbociclib ADME processes. Reduced function of P-gp and CYP3A, as assessed through DDGI, can lead to increased concentrations of palbociclib in the bloodstream. Created with BioRender.com.

Figure 8 summarizes our findings on the crucial roles of P-gp and CYP3A in the ADME processes involving palbociclib. Reduced function of P-gp and CYP3A, as assessed by evaluating DDGIs, may contribute to increased concentrations of palbociclib in the systemic circulation.

Our study faced limitations due to its relatively small sample size and descriptive nature, which restricted our capacity to fully elucidate the clinical significance of the observed palbociclib trough concentration variations. A more comprehensive analysis, potentially incorporating clinical outcomes, would be necessary to fully understand the implications of these pharmacokinetic variations. From a clinical perspective, higher palbociclib C_trough_ values, as indicated by the higher percentage deviations in our results, are expected to increase toxicity, particularly neutropenia. Although increased exposure seems to be associated with a greater reduction in ANC, the data on efficacy, such as progression-free survival, remain inconclusive (Flaherty et al., 2012; Center for Drug Evaluation and Research, 2014). Therefore, future research should prioritize not only validating our findings in larger cohorts but also exploring whether the patient-specific factors we investigated are implicated in the mechanisms underlying these exposure–toxicity associations. This will help to refine palbociclib dosing guidelines to manage toxicity more effectively.

Furthermore, distinct from numerous studies that rely on PK/PD models and population pharmacokinetics from healthy volunteers, our plasma concentration data originate from a real-world cohort of women diagnosed with metastatic breast cancer and undergoing treatment with palbociclib.

## 5 Conclusion

In conclusion, our study offers an in-depth perspective of the pharmacokinetic variability of palbociclib, highlighting the importance of considering a spectrum of factors such as age, renal function, genetic polymorphisms, and concomitant medication in tailoring treatment for patients with HR+/HER2- metastatic breast cancer. It notably highlights the impact of subclinical factors, including variations in creatinine levels and eGFR, or the involvement of moderate to weak inhibitors, on the patient-specific deviations from the median C_trough_ values. This research contributes to the development of more personalized treatment strategies, with the goal of optimizing therapeutic outcomes for each patient undergoing palbociclib treatment for metastatic breast cancer.

## Data Availability

The original contributions presented in the study are included in the article and Supplementary Material, further inquiries can be directed to the corresponding authors.
