# Supplementary material for: Exploring pharmacokinetic variability of palbociclib in HR+/HER2- metastatic breast cancer: a focus on age, renal function, and drug–gene interactions
Source: Front Pharmacol. 2024 Sep 6;15:1420174. doi: 10.3389/fphar.2024.1420174 (PMC11412846; doi:10.3389/fphar.2024.1420174)
Supplement: Supplementary file 1 [file Table1.docx]

***Supplementary Material***

**Supplementary Table 1.** Description and frequency of genotypes found by polymorphism analysis

| Gene | SNP | call rate (%)  n= 68 | Wild type | Heterozygote | Homozygote | Wild type  n (%) | Heterozygote  n (%) | Homozygote  n (%) |
| --- | --- | --- | --- | --- | --- | --- | --- | --- |
| *CYP3A4* | rs35599367 | 100 | C:C | C:T | T:T | 63 (92.6) | 5 (7.4) | 0 (0.0) |
| *CYP3A5* | rs776746 | 100 | A:A | A:G | G:G | 1 (1.4) | 8 (11.8) | 59 (86.8) |
|  | rs10264272 | 100 | C:C | C:T | T:T | 68 (100.0) | 0 (0.0) | 0 (0.0) |
|  | rs41303343 | 100 | -:- | A:- | AA | 68 (100.0) | 0 (0.0) | 0 (0.0) |
| *ABCB1* | rs1128503 | 100 | C:C | C:T | T:T | 22 (32.4) | 34 (50.0) | 12 (17.6) |
|  | rs1045642 | 100 | C:C | C:T | T:T | 20 (29.4) | 32 (47.1) | 16 (23.5) |
|  | rs2032582 | 100 | G:G | G: T/G:A | T:T/A:A/T:A | 20 (29.4) | 33 (48.5) | 15 (22.1) |
| *ABCG2* | rs2231142 | 100 | C:C | C:A | A:A | 54 (79.4) | 14 (20.6) | 0 (0.0) |
| *POR* | rs1057868 | 95.6 | C:C | C:T | T:T | 33 (50.8) | 28 (43.1) | 4 (6.1) |
| *PPARA* | rs4253728 | 98.5 | G:G | G:A | A:A | 35 (51.5) | 23 (33.8) | 10 (14.7) |
|  | rs4823613 | 98.5 | A:A | A:G | G:G | 35 (51.5) | 23 (33.8) | 10 (14.7) |
| *PXR* | rs2472677 | 98.5 | T:T | T:C | C:C | 26 (38.8) | 28 (41.8) | 13 (19.4) |
|  | rs3814055 | 98.5 | C:C | C:T | T:T | 28 (41.8) | 31 (46.3) | 8 (11.9) |
| *VDR* | rs11568820 | 83.8 | C:C | C:T | T:T | 33 (57.9) | 18 (31.6) | 6 (10.5) |
|  | rs1544410 | 83.8 | C:C | C:T | T:T | 26 (45.6) | 27 (47.4) | 4 (7.0) |
|  | rs4516035 | 83.8 | T:T | T:C | C:C | 20 (35.1) | 26 (45.6) | 11 (19.3) |
